# Relating quanta conservation and compartmental epidemiological models of airborne disease outbreaks in buildings

**DOI:** 10.1038/s41598-023-44527-3

**Published:** 2023-10-13

**Authors:** Samuel G. A. Wood, John Craske, Henry C. Burridge

**Affiliations:** Department of Civil and Environmental Engineering, Imperial College London, London, SW7 2AZ UK

**Keywords:** Civil engineering, Epidemiology

## Abstract

We investigate the underlying assumptions and limits of applicability of several documented models for outbreaks of airborne disease inside buildings by showing how they may each be regarded as special cases of a system of equations which combines quanta conservation and compartmental epidemiological modelling. We investigate the behaviour of this system analytically, gaining insight to its behaviour at large time. We then investigate the characteristic timescales of an indoor outbreak, showing how the dilution rate of the space, and the quanta generation rate, incubation rate and removal rate associated with the illness may be used to predict the evolution of an outbreak over time, and may also be used to predict the relative performances of other indoor airborne outbreak models. The model is compared to a more commonly used model, in which it is assumed the environmental concentration of infectious aerosols adheres to a quasi-steady-state, so that the the dimensionless quanta concentration is equal to the the infectious fraction. The model presented here is shown to approach this limit exponentially to within an interval defined by the incubation and removal rates. This may be used to predict the maximum extent to which a case will deviate from the quasi steady state condition.

## Introduction

Airborne transmission plays a central role in the spread of many respiratory illnesses^1^, and typically occurs indoors. Infectious individuals produce droplets laden with infectious material by coughing, sneezing and exhalation; larger droplets can evaporate mid-air, losing mass until they are reduced to a nucleus small enough to be transported as an aerosol by the motion of the air^2^. In this way, infectious material can be spread and, particularly in poorly ventilated spaces, airborne material may linger for long durations resulting in exposures even after infectious individuals have left^3^.

The concentration of infectious material present in an indoor space may be used to predict the likelihood that a susceptible person will become infected^4^. One common method of finding the concentration of infectious aerosol is to treat the air within each indoor space as possessing spatially uniform properties—the so called ‘well-mixed’ assumption. This assumption can then be used in conjunction with knowledge of the number of infectious individuals present, and the rate at which they produce infectious aerosol, to calculate the expected spread of infection over time^3,5^. In such models a number of assumptions are, often implicitly, made about the timescales over which various processes such as dilution, incubation, and the removal of the infectious occur; however, little work exists examining the underlying timescales of these processes and how their relative magnitudes affect the performance of outbreak models.

There are a number of studies in which a conservation equation for the concentration of airborne infectious material is incorporated into epidemiological models to predict outbreaks of different diseases within indoor spaces^6,7^. However, in contrast to the dynamics of population scale epidemiological models^8,9^, the fundamental epidemiological properties of such outbreak models have not been studied extensively.

Many epidemiological models exist in which infection spreads via the environment, rather than directly between infectious and susceptible individuals. In such models, infectious individuals contribute to an environmental pool of infectious material, which may linger for long periods even when no infectious material is present. Typically, these models consider environmental contaminants that may remain for relatively long time periods, for example to predict inter-seasonal transmission of avian flu from contaminated breeding grounds where infectious material remains in faecal matter for many months^10^. In humans, such models are commonly used for longer-lasting environmental contamination such as that seen in water-borne illness^11,12^. There is little existing work that considers the air within an indoor space to be such as an environmental pool. The time over which airborne infectious material lingers is generally considered to be short (due to ventilation, deposition and decay), relative to the timescales of occupation of buildings, and to the periods of incubation and recovery or removal of infectious individuals^13^.

In this work we incorporate a conservation equation for the concentration of airborne infectious material in an indoor space in to a compartmental epidemiological model following a method previously used by Noakes et al.^7^, resulting in an environmental pool model. We consider the resulting system specifically in the context of indoor airborne outbreaks. We study the behaviour of the system, including the timescales of dilution, exposure, incubation and removal. We show how several existing outbreak models may be shown as limiting cases of the resulting system, and how values derived from the combined system may be used to predict the performances of these models relative to each other under different circumstances.

## Quanta-based transmission models and epidemiological modelling

In this section we detail two broad (and sometimes overlapping) classes of model used for outbreaks of infectious disease and their applications to indoor spaces: ‘quanta-based infection models’ which model the spread of infection via airborne ‘quanta’, and ‘compartmental epidemiological models’ which model the spread of disease throughout a population. We discuss their current applications with emphasis on the ways in which they can be brought together.

### Quanta conservation models

For infectious diseases, a complete representation of the infection process requires considered parametrisation of the processes of the production of, and exposure to, infectious aerosols (the dose), as well as the process of in-host infection (the response). Following this modelling approach, these so-called ‘dose-response’ models lead to a more complete representation of this physical process. However, this comes at the cost of the challenge of robust parametrisation^14^. The approaches that we go on to document herein could be simply extended and applied to dose-response models; however, one would be required to specify a particular model for the dose, and for the response, and hence one would be required to focus one a more narrow class of disease. For this reason, we choose to focus on quanta, rather than dose-response, based models of airborne infection.

The ‘quantum’ or ‘quanta’ is a commonly used concept to classify the transmission and infection properties of a given disease^4^, which simplifies the modelling at the expense of providing less complete representation of the infection process. If environmental interactions are such that the infection process follows a Poisson relation, then the exposure to one quantum of infectious material gives an average probability *p* of $p= 1 - e^{-1} \approx 63\%$ of becoming infected^4,15^. The relative simplicity, and more ready ability to parameterise infections based on observed data, has led to quanta-based infection models being widely used for a range of different infection mechanisms, including fomite^16^, droplet and aerosol-borne illnesses^17^.

For airborne outbreaks, if the air within an indoor space is assumed to be ‘well-mixed’, i.e. of spatially uniform concentrations, then considerations regarding the spatial location of individuals either emitting or inhaling quanta are neglected. Additionally, this allows the rate at which quanta leave the space, through a variety of dilution processes, to be more simply modelled. In such indoor spaces, of volume denoted *V*, the quanta concentration, *C* (per unit volume), evolves according to a balance between source and dilution terms.

The source term is the product of the quanta generation rate, *q*, and the number of infectious individuals, ${\hat{I}}$. Appropriate values for the quanta generation rate depend on a wide range of factors, such as the specific disease under consideration, the vulnerability of the population, the emission rate specific to the individual and their activity level (which will also vary over the course of infection), hygiene habits, masking, and the environment conditions (e.g., humidity, temperature, etc.). However, it is common to treat the quanta generation rate as constant for a given outbreak.

The dilution terms of quanta (in some part representing infectious particles) are typically taken to depend on the current quanta concentration, *C*, and the rate of any number of removal mechanisms. The most ubiquitous of which is the rate at which incoming air, carrying no infectious aerosols, is brought within the space, i.e., the ventilation rate $Q_v$. In addition, removal mechanisms can include deposition, decay, and air cleaning and filtering processes^13,18^, with first-order removal rates denoted $\lambda _d$ for deposition, $\lambda _k$ for decay, and $\lambda _c$ for cleaning/filtering processes. This provides a total dilution rate, $Q = Q_v +V(\lambda _d+\lambda _k+\lambda _c)$, providing the quanta conservation equation.

1 $$\begin{aligned} V\,\frac{\text {d}C}{\text {d}t} = {\hat{I}}\,q-Q_v\, C-V \, C\,(\lambda _d + \lambda _k + \lambda _c) = {\hat{I}}\,q - Q\, C. \end{aligned}$$

Perhaps the most commonly used model for predicting the spread of airborne infection in indoor spaces is the ’Wells–Riley’ model. This assumes that the number of infectious individuals within a space remains constant and that they produce infectious aerosol at a constant rate, and that the concentration of infectious material has reached a steady state within the environment, i.e. that infectious aerosol is being removed via dilution at the same rate it is being generated. It is also assumed that the air within the space is well-mixed, so that spatial considerations may be neglected. In this case, the number of individuals exposed (infected but not actively producing infectious aerosols) will be given over a time interval by^19^

2 $$\begin{aligned} {\hat{E}} = {\hat{S}} \left( 1-e^{ - \frac{ {\hat{I}} q p}{Q} t } \right) \;, \end{aligned}$$

where ${\hat{E}}$ is the size of the population exposed after time *t* since the onset of the outbreak, ${\hat{S}}$ is the size of the initially susceptible population, and *p* is the pulmonary respiration rate. The Wells-Riley model is valid when both the time frame of the outbreak under investigation is short relative to the incubation period of the disease, and when the quasi steady state is reached rapidly^13^.

A variant of the Wells–Riley model, in which the assumption of a steady state quanta concentration is relaxed, is provided by the Gammaitoni-Nucci model^3^. In this model, an equation for the conservation of quanta is necessary to model the effects of changes in infectious aerosol concentration over time. The Gammaitoni-Nucci model couples (1) with

3 $$\begin{aligned} \frac{\text {d}{\hat{E}}}{\text {d}t}=-\frac{\text {d}{\hat{S}}}{\text {d}t}=C\,p\,{\hat{S}}, \end{aligned}$$

and solves these coupled equations, implicitly assuming that the time frame of the outbreak is short relative to the incubation period of the disease.

### Compartmental epidemiological models

The use of compartmental epidemiological modelling has been applied to the outbreaks of infectious diseases dating back to at least as far as the early twentieth century^20^. Such models segregate the population into ‘compartments’, defined by distinct stages of the infection process, and track the evolution of these population compartments over the course of the outbreak^8^.

The SEIR model is a widely used compartmental epidemiological model^1^, in which a system of differential equations are used to describe an outbreak. The equations track the number of susceptible individuals (${\hat{S}}$), the number of individuals who have been exposed to a pathogen but are not yet infectious themselves (${\hat{E}}$), the number who are infectious (${\hat{I}}$), and the number removed (${\hat{R}}$, i.e. those who may have died, developed immunity, or been removed from the population, e.g. via isolation). For a given population of size *N*, ${\hat{S}}+{\hat{E}}+{\hat{I}}+{\hat{R}} = N$, making it convenient to employ scaled population variables, *S*, *E*, *I*, *R*:

4 $$\begin{aligned} {\hat{S}}/N+{\hat{E}}/N+{\hat{I}}/N+{\hat{R}}/N=S+E+I+R = 1. \end{aligned}$$

As is common with epidemiological models, all population variables are approximated to be continuous; an approximation of little consequence for sufficiently large populations.

The scaled population variables evolve according to the following ordinary differential equations (ODEs),

5a $$\begin{aligned} \frac{\text {d}S}{\text {d}t}=-\beta \, S \, I , \end{aligned}$$

5b $$\begin{aligned} \frac{\text {d}E}{\text {d}t}=\beta \, S \, I-\omega E,\end{aligned}$$

5c $$\begin{aligned} \frac{\text {d}I}{\text {d}t}=\omega E -\gamma I ,\end{aligned}$$

5d $$\begin{aligned} \frac{\text {d}R}{\text {d}t} =\gamma \, I, \end{aligned}$$

where $\beta$ is the ‘contact rate’ between susceptible and infectious individuals, $\omega$ is the rate at which exposed individuals become infectious and $\gamma$ is the rate at which infectious individuals are removed. Note that as $S+E+I+R=1$ is a conserved quantity, these are not independent equations.

The SEIR model assumes that the spread of disease may be represented solely through interactions between infectious and susceptible individuals with the parameter $\beta$, which accounts for the frequency and nature of these interactions, as well as for the infectiousness of the particular pathogen. As such, the SEIR model provides no mechanism for infection to occur via exposure to infectious material that remains within an environment in the absence of infectious people, as can occur in the case of both fomite and airborne transmission.

The SEIR model is a longstanding model^21^ that has been extensively studied and deployed to understand and respond to outbreaks of numerous diseases including influenza^22^, ebola^23^, tuberculosis^24^, measles^25^, mumps^26^. More recently, the SEIR model has been useful in the study of COVID-19^27,28^, playing a significant role in informing the response to COVID-19, and has even been used by news agencies to support the communication of the response to the public^29^. The relative simplicity of SEIR-type models has allowed them to be effectively integrated with other models, such as mobility network models which track the movement of populations in an urban environment in order to identify the locations in which an outbreak is likely to spread^30^. It is noteworthy that many of the diseases for which the SEIR model has been deployed are airborne, including COVID-19.

There exist some overlaps between quanta-based modelling and compartmental epidemiological models. For example, the Gammaitoni-Nucci model, see (3), may be regarded as a highly simplified compartmental model in which the population may only move from susceptible to exposed, under the governance of the quanta conservation equation.

## The SEIR equations with airborne infectious material

For airborne pathogens, infection is spread not by direct interactions between susceptible and infectious individuals, but by the inhalation of infectious aerosols. Therefore, following Noakes et al.^7^ and Gammaitoni & Nucci^3^, we modify the SEIR equations to be suitable for modelling the transmission of airborne diseases by writing the (negative) growth rate of the susceptible population as the product of the population size, *S*, the concentration, *C*, of infectious aerosol (in our case, represented by quanta), and the rate at which susceptible individuals breathe in this air, as determined by their pulmonary breathing rate, *p*.

Incorporating (1) into the SEIR model yields a system of five coupled first order ODEs, which account for the effects of environmental transients within outbreaks of airborne disease. We describe this as the ‘SEIR-C’ system. Here, ’C’ refers to the addition of a conservation equation for the infectious quanta within the environment, *C*, which couples with the other equations by affecting the rate of change of the susceptible population, but ‘C’ is not itself a population compartment.

Within the SEIR-C system, the quanta concentration and the fractions of the population at each stage of the infection process evolve according to

6a $$\begin{aligned} V\frac{\text {d}C}{\text {d}t} = N \,I\,q-Q\,C ,\end{aligned}$$

6b $$\begin{aligned} \frac{\text {d}S}{\text {d}t}=-C\,p\,S , \end{aligned}$$

6c $$\begin{aligned} \frac{\text {d}E}{\text {d}t}=C\,p\,S-\omega \,E ,\end{aligned}$$

6d $$\begin{aligned} \frac{\text {d}I}{\text {d}t}=\omega \, E -\gamma \,I ,\end{aligned}$$

6e $$\begin{aligned} \frac{\text {d}R}{\text {d}t} =\gamma \,I . \end{aligned}$$

**Figure 1:**
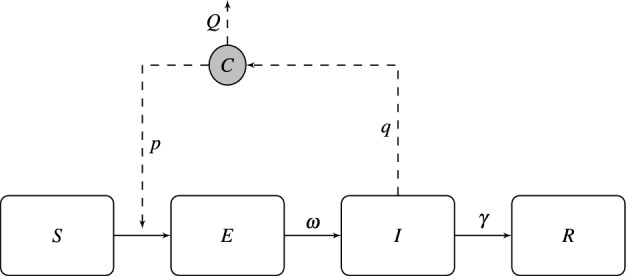
Summary of the SEIR-C system showing the four states an individual can take and the parameters that govern the transitions between them, as well as the interaction with the airborne quanta concentration *C*.

A schematic illustration of this system is shown in Fig. 1

### The dimensionless SEIR-C system

Here we identify a minimal set of dimensionless parameters which determine the response of the SEIR-C system.

We use the volume of the room environment, *V*, and dilution rate within it, *Q*, to introduce the dilution timescale

7 $$\begin{aligned} T_d=\frac{V}{Q}, \end{aligned}$$

and define the dimensionless time

8 $$\begin{aligned} \tau =\frac{t}{T_d}, \end{aligned}$$

and the scaled incubation and removal rates

9 $$\begin{aligned} \Omega = \omega \, T_d \;\; \text {and} \;\; \Gamma = \gamma \, T_d, \end{aligned}$$

respectively.

Finally, we scale *C* such that the dimensionless quanta concentration is

10 $$\begin{aligned} \eta = \frac{C\,Q}{N\,q}, \end{aligned}$$

to yield the system of dimensionless equations as

11a $$\begin{aligned} \frac{\text {d}\eta }{\text {d}\tau }=I-\eta , \end{aligned}$$

11b $$\begin{aligned} \frac{\text {d}S}{\text {d}\tau } = -\alpha \, \eta \, S ,\end{aligned}$$

11c $$\begin{aligned} \frac{\text {d}E}{\text {d}\tau } = \alpha \, \eta \, S -\Omega \, E ,\end{aligned}$$

11d $$\begin{aligned} \frac{\text {d}I}{\text {d}\tau }=\Omega \, E - \Gamma \, I ,\end{aligned}$$

11e $$\begin{aligned} \frac{\text {d}R}{\text {d}\tau }=\Gamma \, I , \end{aligned}$$

where

12 $$\begin{aligned} \alpha = \frac{V N p q}{Q^2}. \end{aligned}$$

The dimensionless system is therefore characterised by three dimensionless parameters, $\{\alpha , \Omega , \Gamma \}$. The parameter $\Omega$ represents the ratio of the disease incubation period and the dilution timescale $T_d$, similarly $\Gamma$ the ratio of the average infectious period and $T_d$.

The parameter $\alpha$ may be considered in terms of the timescales $T_q = 1/q$, which we describe as the quanta timescale, and $T_p = V/(p \, N)$, which we describe as the filling timescale—note the inclusion of the factor *N* within $T_p$ in order that this timescale is the filling timescale associated with all occupants breath within the room. The parameter $\alpha$ is the product of the ratios of the dilution timescale to the filling timescale, and that of the dilution timescale to the quanta timescale.

13 $$\begin{aligned} \alpha = \frac{T_d}{T_q} \frac{T_d}{T_p}. \end{aligned}$$

To aid interpretation of this SEIR-C system, it is helpful to consider the physical relevance of the dependent variable $\eta$. Firstly, consider the special case in which the environment is in a steady state, for which (11a) reduces to

14 $$\begin{aligned} \eta _{ss} = I \,. \end{aligned}$$

This highlights that it can be useful to consider the dimensionless quanta concentration, $\eta$ via the ratio $\eta /I$, that is the ratio of the current quanta concentration to that which would be obtained in steady state. Hence, all else remaining equal, as time evolves the ratio $\eta / \eta _{ss}$ will approach unity either from above or below. Similar insight is provided by considering $\eta$ as the ratio of the current removal rate, $C\,Q$, to the maximum quanta generation rate if all occupants were infectious, $N\,q$.

### Deterministic and stochastic models

The deterministic nature of the SEIR-C system, and the models that can be extracted from it, may limit their applicability, particularly when the population size is small. The difference between stochastic and deterministic models in epidemiology has been extensively studied, for example for susceptible-infectious models^31^ and for SIR models^32^.

Deterministic models will still give useful indications of the outbreak behaviour in such cases, particularly when comparing the effects of infection control measures, hence the widespread use of the Wells-Riley model and deterministic models like SEIR^7^. Additionally, it is possible to formulate equivalent stochastic models from the deterministic system, if required. Although analysis of the deterministic SEIR-C system is the focus of this work, we present a basic approach to building an equivalent stochastic model formulated from the deterministic system.

The probability of exposure for an individual can be expressed as a function of the total dose *D* received, which in the dimensionless system is

15 $$\begin{aligned} P_{E} = 1- \exp ^{-D} = 1- \exp ^{-\alpha \int {\eta \text {d}\tau }} \,, \end{aligned}$$

and

16 $$\begin{aligned} \frac{\text {d}D}{\text {d}\tau } = \alpha \eta \,. \end{aligned}$$

This may be used to predict the per-susceptible possibility of exposure associated with a time step with a chosen numerical method, alongside a per-exposed possibility of incubation, and a per-infectious possibility of removal based on the incubation and removal rates, and from this a stochastic model may be formulated, for example using Eulers method. Similar approaches have been used in the past for the SEIR^33^ and Wells–Riley^5^ models.

The above approach is useful for considering stochastic effects that arise from small populations, namely the possibility of extinction in the early stages of an outbreak, but there are many different approaches to stochastic modelling of epidemiological models which may be applied to account for a wide range of different effects. For example, it is possible to randomly vary the incubation or removal rates within the population according to a distribution, or to vary the production-dilution parameter to account for the extreme variation in concentration of infectious material seen in some environments^34,35^, as real outbreak data has often been shown to be significantly overdispersed compared to deterministic models due to high variation in individual infectiousness^36^, though such variations are beyond the scope of the current study.

### Recovering quanta-based and epidemiological infection models from the SEIR-C system

#### Gammaitoni-Nucci and Wells-Riley models

The classical Wells-Riley airborne infection model, and the Gammaitoni-Nucci variant, discussed in “Quanta conservation models”, may be obtained from the SEIR-C system under the assumption that the exposed never become infectious themselves, and that the infectious are never removed, i.e. $\Omega = 0$ and $\Gamma = 0$—or that the rate the incubation and removal timescales are sufficiently large relative to the outbreak duration that the effects of these processes are negligible. Doing so is sufficient to recover the Gammaitoni-Nucci model but to recover Wells-Riley one is further required to assume that the airborne quanta concentration is always at equilibrium such that $\eta =\eta _{ss} = I$, at all times.

#### SEIR epidemiological model

A special case of the SEIR-C system occurs when the rate at which occupants become infectious, $\text {d}I / \text {d}t$, is small relative to the rate over which the quanta concentration becomes steady. Only in such cases is it reasonable to assume that the quanta concentration takes the value associated with the concentration at steady state, i.e. $\text {d}C / \text {d}t =0$, and the steady state quanta concentration

17 $$\begin{aligned} C_{ss}=\frac{q\,N \, I}{Q} \,, \end{aligned}$$

is adhered to at all times. Equivalently, in the dimensionless system, (11), $\eta =\eta _{ss} = I$.

We describe this special case as the ‘quasi-steady-state assumption’. Applying this assumption (i.e. $\text {d}C / \text {d}t = 0$) and substituting (17) into (6b) highlights that the standard form of the SEIR model can be recovered from the SEIR-C system but only in the special case that the quasi-steady-state condition is met and one arbitrarily sets the product of the quanta generation rate, *q*, and the ratio of the pulmonary breathing rate and the dilution rate, *p*/*Q*, to be the contact rate, $\beta$. Note that in the SEIR-C system, exposures arise not from direct contact between susceptible and infectious individuals but from the exposure of susceptibles to airborne infectious material and so any analogy regarding the ‘contact rate’ is purely mathematical.

## Analysis of the SEIR-C system

We now highlight three pertinent research questions regarding the behaviour of the SEIR-C system, which are answered in this section. Firstly, the SEIR-C system (as with the SEIR model), by including the concept of removal, does not require the whole population to become infected, irrespective of the length of the outbreak considered. Hence, it is important to establish the fraction of susceptibles that will remain at large time, $S_f$, since this is the fraction of the population who will avoid the disease. We determine the analytical solution for $S_{f}$ in “The SEIR-C system at large time”. Secondly, the time frame over which infection spreads is pertinent when trying to manage outbreaks, and is also an important consideration when selecting an appropriate outbreak model for a given context. In “Timescales in the SEIR-C system” we identify the dominant timescales relating to the different stages of an outbreak. Finally, as discussed in “Recovering quanta-based and epidemiological infection models from the SEIR-C system”, the quasi-steady-state condition is maintained, the SEIR-C system is mathematically similar to the SEIR model. As such the relationship between the actual dimensionless quanta concentration, $\eta$, and that which would be predicted under the quasi-steady-state assumption $\eta _{ss}=I$ is investigated to highlight regimes for which the simplifying assumption of the quasi-steady state is valid in “The quasi-steady-state assumption”.

### The SEIR-C system at large time

We seek an expression for the fraction of susceptibles ultimately remaining at large time, denoted $S_f$. It has been shown^8^, for both the SIR and SEIR model, that for a given contact rate $\beta$, an analytic solution exists for this fraction, and is given by the implicit equation

18 $$\begin{aligned} S_f-\frac{\gamma }{\beta }\ln (S_f)=-\frac{\gamma }{\beta }\ln (S_0) +1. \end{aligned}$$

A similar approach may be taken for the extended SEIR-C system. Adding (11b), (11c) and (11d), writing $F=S+E+I$, and using the chain-rule and (11b) to re-express derivatives with respect to $\tau$ in terms of *S* gives

19 $$\begin{aligned} \frac{\text {d}F}{\text {d}S}=\frac{\Gamma }{\alpha S} \frac{I}{\eta }, \end{aligned}$$

and hence

20 $$\begin{aligned} \frac{\text {d}F}{\text {d}\ln (S)}=\frac{\Gamma }{\alpha } \frac{I}{\eta }, \end{aligned}$$

giving

21 $$\begin{aligned} F_\tau -F_0=\frac{\Gamma }{\alpha } \int ^{\ln (S_f)}_{\ln (S_0)}\frac{I}{\eta }\; \; \text {d}\!\ln (S). \end{aligned}$$

To evaluate the right hand side of (21), (11a) may be used:

22 $$\begin{aligned} \int _0^\tau \frac{\text {d}\eta }{\text {d}\tau } \; \; \text {d}\tau = \int _0^\tau \eta \left( \frac{I}{\eta }-1\right) \text {d}\tau , \end{aligned}$$

and $\eta$ substituted from (11b) to give

23 $$\begin{aligned} \eta _\tau -\eta _0= -\frac{1}{\alpha }\int _0^\tau \frac{\text {d}\!\ln (S)}{\text {d}\tau }\left( \frac{I}{\eta }-1\right) \text {d}\tau . \end{aligned}$$

Since *S* varies monotonically with $\tau$, such that $\frac{\text {d}\!\ln (S)}{\text {d}\tau }\text {d}\tau =\text {d}\ln (S)$, then

24 $$\begin{aligned} \int _{\ln (S_0)}^{\ln (S_f)}\frac{I}{\eta } \; \; \text {d}\!\ln (S)= \int _{\ln (S_0)}^{\ln (S_f)}\; \; \text {d}\!\ln (S)-\alpha (\eta _\tau -\eta _0), \end{aligned}$$

and (21) becomes

25 $$\begin{aligned} S_f+E_\tau +I_\tau -S_0-E_0-I_0=\frac{\Gamma }{\alpha }(\ln (S_f)-\ln (S_0))-\Gamma (\eta _\tau -\eta _0). \end{aligned}$$

Noting that $\eta _\tau$, $E_\tau$ and $I_\tau$ all tend to zero over large time and, without loss of generality taking $R_0=0$, the initial fractions of susceptibles, exposed and infectious must account for the entire population so that $S_0+E_0+I_0=1$ giving

26 $$\begin{aligned} S_f-\frac{\Gamma }{\alpha }\,\ln (S_f)=-\frac{\Gamma }{\alpha }\,\ln (S_0)+\Gamma \, \eta _0+1. \end{aligned}$$

This result provides the means to evaluate $S_f$, and demonstrates two important properties of the system. Firstly, when $\eta _0=0$ (i.e. when there is no infectious aerosol initially present in the environment), the final state of the system depends solely on the ratio of the removal rate to the production-dilution parameter, $\Gamma /\alpha$. When $\eta _0\ne 0$, the values of $\alpha$ and $\Gamma$ influence the end state independently of one another. Secondly, it can be seen that the final state of the model is completely independent of the dimensionless incubation rate $\Omega$, as is also the case for the SEIR model^8^. Taking the initial dimensionless quanta concentration to be zero, and comparing (26) to the equivalent expression for the SEIR model, i.e. (18), is is clear that the fraction of remaining susceptibles over large time in the SEIR-C system takes a similar form as that for the SEIR model.

From (26), and again taking cases for which there is no infectious aerosol initially present ($\eta _0=0$), the ultimate state of the outbreak can be determined from

27 $$\begin{aligned} \frac{\Gamma }{\alpha }=\frac{1-S_f}{\ln (S_0)-\ln (S_f)}. \end{aligned}$$

In such cases, if $\Gamma /\alpha$ is large, $S_f$ must approach the value of $S_0$, implying that the outbreak will be effectively shut down. Similarly, as $S_f\le S_0$ (due to the monotonic nature of *S*), and $S_0 = 1-I_0$ where $I_0$ is typically small, then if $\Gamma /\alpha$ is small, then too, $S_f$ must be small, implying that much of the population will become infected.

### Timescales in the SEIR-C system

Although (26) predicts the eventual state of the system, it provides no information about the timescales over which outbreaks might occur. Solutions that uniquely define the duration of outbreaks for the classical SEIR model remain elusive^37^, and this is case for the SEIR-C system too. However, we go on to present a method to identify a number of characteristic timescales which can each play a role in determining outbreak durations. These are presented as timescales in the dimensionless system, i.e. physical timescales that have been normalised by the timescale $T_d=V/Q$ to render them dimensionless.

The physics of the system indicate that all three parameters might influence the outbreak time; namely, the production-dilution parameter $\alpha$ influencing the rate at which new exposures occur, the incubation period $\Omega$ acting as a lag period before infectiousness, and the rate of removal $\Gamma$ limiting the period of infectiousness. The role of each of these parameters may be explained by an understanding of the (dimensionless) timescales which emerge in the dimensionless model. Four different timescales are considered, each of them associated with a physical process. The first is associated with exposures due to the initially present infectious aerosol, which we describe as the initial-exposure timescale and denote ${\mathscr {T}}_{\eta }$. Another is associated with the production of quanta, and the resultant exposures, in the infectious population, which we describe as the production-exposure timescale, and denoted ${\mathscr {T}}_E$. A third is associated with the incubation period, which we refer to as the incubation timescale, ${\mathscr {T}}_\Omega$, and the final timescale is associated with the removal period, and is denoted the removal timescale ${\mathscr {T}}_\Gamma$.

The initial-exposure timescale ${\mathscr {T}}_\eta$ is considered first. The fraction of the population who are exposed only through the initially present infectious aerosol may be considered by analysis of the system when two conditions are met: the incubation rate is considered to be small ($\Omega \approx 0$), so that those primary exposures never become infectious themselves, and the fraction of infected initially present is treated as zero, i.e. $I_0$ = 0, so that the susceptible fraction *S* will be influenced only by the primary exposures. These assumptions will provide a good representation of the original system, provided that this timescale is small relative to both the incubation timescale and the exposure timescale that results from quanta generation by the infectious population. Setting $I_0=0$ and integrating (11a) gives

28 $$\begin{aligned} \eta = \eta _0 \, e^{-\tau }, \end{aligned}$$

which, combined with (11b), provides

29 $$\begin{aligned} S = \frac{S_0}{e^{\alpha \, \eta _0}} \, e^{(\alpha \, \eta _0 \, e^{-\tau })}. \end{aligned}$$

When the incubation rate is approximately zero, the fraction of the population that remains susceptible over large time $S_\eta$ is

30 $$\begin{aligned} S_\eta =S_0 \, e^{-\alpha \, \eta _0}. \end{aligned}$$

Considering the initial gradient of *S* gives

31 $$\begin{aligned} \frac{\text {d}S}{\text {d}\tau } \Big |_{\tau =0} = -\alpha \, \eta _0 \, S_0. \end{aligned}$$

The timescale, ${\mathscr {T}}_\eta$ can therefore be defined by

32 $$\begin{aligned} S_\eta = S_0 + \frac{\text {d}S}{\text {d}\tau } \Big |_{\tau =0} {\mathscr {T}}_\eta = S_0 -\alpha \, \eta _0 \, S_0 {\mathscr {T}}_\eta . \end{aligned}$$

Rearranging and using (30), we obtain

33 $$\begin{aligned} {\mathscr {T}}_\eta =\frac{ 1-e^{-\alpha \, \eta _0}}{\alpha \, \eta _0}. \end{aligned}$$

Since $\eta _0$ and $\alpha$ are strictly positive, the timescale ${\mathscr {T}}_\eta$ must lie in the range $0<{\mathscr {T}}_\eta <1$. Physically, this states that the initial-exposure timescale must always be less than the dilution timescale, and the exposures due to the initial infectious aerosol are limited by the rate at which dilution occurs.

Consider now the timescale describing the production of quanta and the resultant exposures, ${\mathscr {T}}_E$, firstly in the absence of any initially present infectious material. This timescale may be identified by use of an approximation for *S* based on the initial condition, and derivatives of the initial condition. It might be desirable to allow a linear approximation to define this timescale; however, the first derivative of *S*, i.e. (11b), is zero when $\eta _0=0$, and hence, a quadratic approximation is used herein (a commonly employed technique; for example, Section 6.4^38^, there used to define the Taylor microscale from the spatial velocity autocorrelation in a turbulent flow field). Differentiating (11b) gives

34 $$\begin{aligned} \frac{\text {d}^2S}{\text {d}\tau ^2}= - \alpha \, S \frac{\text {d}\eta }{\text {d}\tau } - \alpha \, \eta \frac{\text {d}S}{\text {d}\tau } = - \alpha \, S \, (I-\eta )+\alpha ^2 \, \eta ^2 \, S, \end{aligned}$$

and

35 $$\begin{aligned} \frac{\text {d}^2S}{\text {d}\tau ^2} \Big |_{\tau =0}=- \alpha \, S_0 (I_0-\eta _0)+\alpha ^2 \, \eta _0^2 \, S_0. \end{aligned}$$

The exposure production-timescale ${\mathscr {T}}_E$ is introduced (recalling that $\eta _0 = 0$) as satisfying

36 $$\begin{aligned} S_0-S_f = -\frac{\text {d}^2S}{\text {d}\tau ^2} \Big |_{\tau =0} {\mathscr {T}}_E ^2= \alpha \, S_0 \, I_0 \, {\mathscr {T}}_E ^2, \end{aligned}$$

and hence

37 $$\begin{aligned} {\mathscr {T}}_E = \sqrt{\frac{S_0-S_f}{\alpha \, S_0 \, I_0}}, \end{aligned}$$

where $S_f$ is provided by (26).

It was shown in “The SEIR-C system at large time” that, when the production-dilution parameter is large relative to the removal rate, the fraction of susceptibles will ultimately approach zero ($S_f\approx 0$). In these cases, the timescale ${\mathscr {T}}_E$ is given by $\sqrt{1/(\alpha \, I_0)}$.

**Figure 2:**
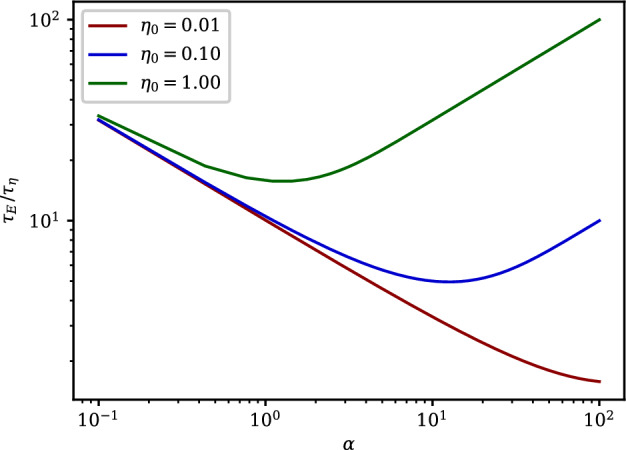
The ratio of the production-exposure, ${\mathscr {T}}_E$ to the initial-exposure timescale ${\mathscr {T}}_\eta$ as a function of $\alpha$, for the case where $I_0 = 0.01$ and $S_f \approx 0$.

Figure 2 shows the ratio of the production-exposure timescale to the initial-exposure timescale as a function of $\alpha$; three different initial quanta concentrations are plotted, in each case the initial infectious fraction is taken to be $I_0=0.01$, and $\Gamma \ll \alpha$ so that the entire susceptible population will become infected ($S_f \approx 0$). It may be seen from the figure that only when there is a small amount of infectious material initially present, and the production-dilution parameter is very large, can ${\mathscr {T}}_E$ be of similar magnitude to ${\mathscr {T}}_\eta$.

Finally, the incubation timescale and removal timescale arise, more naturally, from the incubation and removal periods, respectively. As such, they are simply defined.

38 $$\begin{aligned} {\mathscr {T}}_\Omega = \frac{1}{\Omega } \,, \end{aligned}$$

and

39 $$\begin{aligned} {\mathscr {T}}_\Gamma = \frac{1}{\Gamma } \,. \end{aligned}$$

A summary of the four timescales identified, along with their definitions, is provided in Table 1. Their role in describing the progress of an outbreak and the relative behaviour of different outbreak models is explored further in “Outbreak predictions for the SEIR-C system and existing airborne infection models”.

**Table 1:** The timescales associated with the SEIR-C system and their definitions.

Timescale	Symbol	Definition
Initial-exposure	${\mathscr {T}}_\eta$	$\frac{ 1-e^{-\alpha \, \eta _0}}{\alpha \, \eta _0}$
Production-exposure	${\mathscr {T}}_E$	$\sqrt{\frac{S_0-S_f}{\alpha \, S_0 \, I_0}}$
Incubation	${\mathscr {T}}_\Omega$	$\frac{1}{\Omega }$
Removal	${\mathscr {T}}_\Gamma$	$\frac{1}{\Gamma }$

### The quasi-steady-state assumption

**Figure 3:**
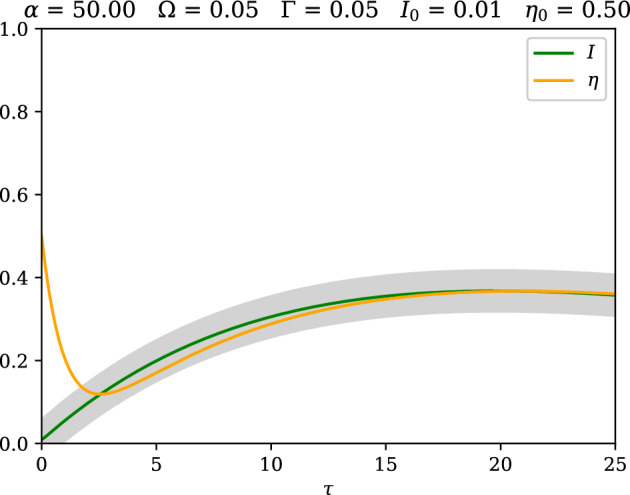
An example case where $\alpha = 50$, $\Omega =0.05$ and $\Gamma =0.05$ with an initial condition of $I_0=0.01$ and $\eta _0 = 0.5$, showing $\eta$ falling into and remaining in the interval defined by $[I-\Omega ,\, I + \Gamma ]$, which is denoted by the shaded region.

The quasi-steady-state condition (i.e. that the dimensionless quanta concentration maintains an approximate equilibrium with the infectious fraction, leading to the assumption that $\eta = I$) has been widely used to predict the evolution of outbreaks of airborne disease by removing the requirement to consider the conservation of quanta^39–41^ as previously discussed in “Recovering quanta-based and epidemiological infection models from the SEIR-C system”. Here, we investigate the limits of validity of the quasi-steady-assumption.

We start by proving that the deviation from the quasi-steady-state condition, (i.e $I-\eta )$, tends to the interval $[-\Gamma ,\Omega ]$ exponentially. First consider the case in which $I-\eta \ge \Omega$, and define $U = I-\eta - \Omega \ge 0$, so that from (11a) and (11d)

40 $$\begin{aligned} \frac{\text {d}U}{\text {d}\tau } = \eta - I + \Omega E - \Gamma I \le -U, \end{aligned}$$

where the inequality arises from expressing the right-hand side of the equation as $-U-\Omega (1-E)-\Gamma I$ and utilising the facts that $\Omega \ge 0$, $\Gamma \le 0$, $E\le 1$ and $I\ge 0$. Gronwall’s lemma for bounding functions satisfying a known differential inequality, such as (40), then implies that *U* tends to zero exponentially^42^, since

41 $$\begin{aligned} U(\tau ) \le \, U(0)\, e^{-\tau }. \end{aligned}$$

Similarly, when $I-\eta \le -\Gamma$, we define $L = \eta - I - \Gamma \ge 0$, such that

42 $$\begin{aligned} \frac{\text {d}L}{\text {d}\tau } = I - \eta - \Omega E + \Gamma I = -L - \Gamma + \Gamma I - \Omega E\le -L, \end{aligned}$$

where this inequality arises from expressing the right-hand side of the equation as $-L-\Gamma (1-I)-\Omega E$ and utilising the facts that $\Omega \ge 0$, $\Gamma \ge 0$, $I\le 1$ and $E\ge 0$; hence

43 $$\begin{aligned} L(\tau ) \le \, L(0) \, e^{-\tau }. \end{aligned}$$

The exponential decay, to zero, of both *U* and *L* implies that all solutions of the governing equations approach the interval defined by $I-\eta \in [-\Gamma ,\Omega ]$ at least exponentially. Alternatively, if $\Delta >\max (\Gamma ,\Omega )$ then $|I-\eta |\le \Delta$ defines an absorbing set. An example of this behaviour for a case where the production-dilution parameter is large, and the initial condition is far from the quasi steady state is seen in Fig. 3, where $\eta$ may be seen to fall rapidly into this interval and remains there. Operationally, for a given application, if it is important that the quasi-steady state be satisfied to within a given tolerance $\Delta$, then this result shows that $\Delta$ must be large relative to both the recovery rate and incubation rate, which are typically small for outbreaks of most known diseases.

For models which do not incorporate incubation or removal (e.g. the Wells-Riley and the Gammaitoni-Nucci models), if the initial condition meets the quasi-steady-state condition, the quasi-steady-state condition will always be met, as $\Gamma = \Omega = 0$.

### Basic reproduction number

The basic reproduction number is the expected number of secondary infections arising per infectious individual introduced into a susceptible population^43^.

In the SEIR-C system the addition of infectious individuals does not lead to more infections via contact. Instead infectious individuals contribute to the amount of infectious material within the environment. As infections caused in this way can not be seen as directly caused by a particular individual, it is not straightforward to define a basic reproduction number. One method is to consider the number of exposures that will occur if a single infectious individual is introduced to a susceptible population^44^ if no more individuals become infectious themselves (ie. assuming an incubation rate $\Omega$ of zero).

With $\Omega = 0$, (11d) may be integrated to yield

$$\begin{aligned} I = I_0 e^{-\Gamma \tau } \,, \end{aligned}$$

This is inserted into (11a) and integrated to give

$$\begin{aligned} \eta = \frac{I_0 e^{-\Gamma \tau }}{1-\Gamma }+ \left( \eta _0-\frac{I_0}{1-\Gamma } \right) e^{-\tau } \,, \end{aligned}$$

which is then inserted into (11b) and integrated to give

$$\begin{aligned} S = S_0 e^{-\alpha (\frac{I_0}{\Gamma (1-\Gamma )} +\eta _0 - \frac{I_0}{1-\Gamma }) } e^{\alpha ( \frac{I_0}{\Gamma (1-\Gamma )} e^{-\Gamma \tau } +(\eta _0 - \frac{I_0}{1-\Gamma }) e^{-\tau } ) \,. } \end{aligned}$$

When $\eta _0 = 0$ , as $\tau$ tends to infinity, the susceptible fraction of the population at large time is given by

$$\begin{aligned} S_f = S_0 e^{-\alpha (\frac{I_0}{\Gamma (1-\Gamma )} - \frac{I_0}{1-\Gamma }) } = S_0 e^{- \frac{ \alpha I_0}{\Gamma } \,. } \end{aligned}$$

The number of secondary infections from an initially infectious population of size $I_0$ therefore depends upon the size of the susceptible population, and the ratio of the production-dilution parameter to the removal rate.

Conventionally, the basic reproduction number is defined for a single infectious individual initially present ($I_0 = \frac{1}{N}$ in the dimensionless system). When $I_0 = \frac{1}{N}1/N$ and $S_0 = 1 - I_0$, and recalling that for large time the fraction of the population removed $R_f = 1- S_f$

$$\begin{aligned} R_f = 1- S_0 e^{- \frac{ \alpha I_0}{\Gamma } } =1- (1-\frac{1}{N}) e^{- \frac{ \alpha }{\Gamma N} } \,. \end{aligned}$$

As $R_f$ is normalised for population, the basic reproduction number $\hat{R_0}$ is given by

44 $$\begin{aligned} \hat{R_0} = N-(N-1)e ^{- \frac{ \alpha }{\Gamma N} } \,. \end{aligned}$$

Note that $\hat{R_0}$ is the convention for basic reproduction number, and does not here denote an initial condition.

It may be seen that, for a given population, $\hat{R_0}$, depends solely on the ratio of the production-dilution parameter to the removal rate. This is consistent with the observation in “The SEIR-C system at large time” that when the ratio $\alpha /\Gamma$ is large, only a small fraction of the population will become infected, and when it is small the entire population is likely to be infected.

## Outbreak predictions for the SEIR-C system and existing airborne infection models

**Figure 4:**
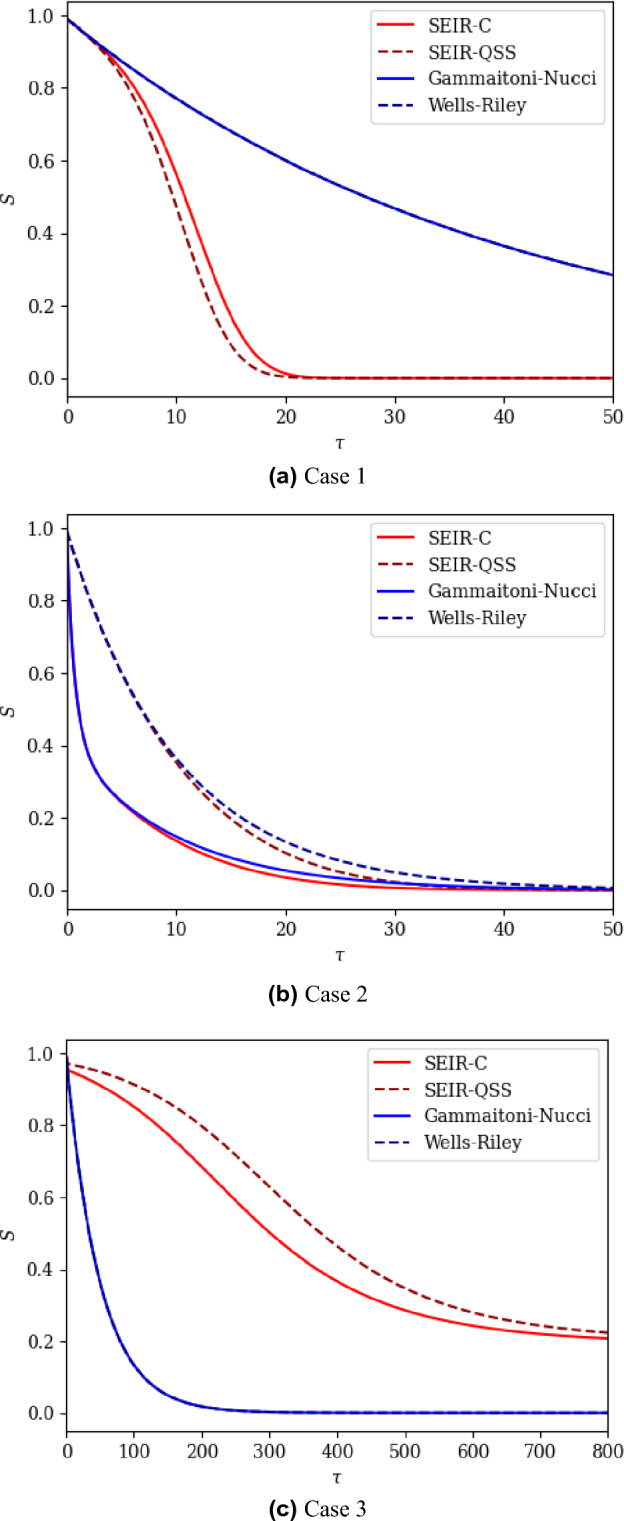
The value of *S* as a function of dimensionless time for the three example outbreak cases described in Table 2.

We now deploy our analysis of the SEIR-C system (“4”) to provide insight as to how the system will behave for three example outbreak cases. We go on to compare the predicted outbreaks to those predicted by three widely used models of outbreaks of airborne disease, selecting the three models which we recovered as limiting cases of the SEIR-C system in “Recovering quanta-based and epidemiological infection models from the SEIR-C system”. Table 2 presents these example cases, in which values of relevant parameters and initial conditions have been selected; the table includes key values from, and timescales in, the SEIR-C system. By considering these timescales, alongside other theory presented in “Analysis of the SEIR-C system”, insight is gained into the behaviour of the SEIR-C system, and of the other airborne infection models. For these three cases, we present the results for the SEIR-C system, and compare them to: the SEIR model recovered by imposing the quasi-steady-state-condition (referred to here as ‘SEIR-QSS’), the Gammaitoni-Nucci model, and the classical Wells–Riley model, in Fig. 4.

**Table 2:** The parameters and initial conditions defining three outbreak cases, key values that can be derived from these, and the timescales that result from these in the SEIR-C system.

Case	Parameters	Initial condition	Derived values	Timescales
1	$\alpha = 2.5$ $\Omega = 0.04$ $\Gamma = 0.008$	$\eta _0 = 0.01$ $S_0 = 0.99$ $E_0 = 0$ $I_0 = 0.01$	$\alpha / \Gamma = 25$ $S_f = 0$ $S_\eta = 0.98$	${\mathscr {T}}_\eta = 0.988$ ${\mathscr {T}}_E$ = 6.325 ${\mathscr {T}}_\Omega = 25$ ${\mathscr {T}}_\Gamma = 125$
2	$\alpha = 10$ $\Omega = 0.0004$ $\Gamma = 0.004$	$\eta _0 = 0.1$ $S_0 = 0.99$ $E_0 = 0$ $I_0 = 0.01$	$\alpha / \Gamma = 2500$ $S_f = 0$ $S_\eta = 0.36$	${\mathscr {T}}_\eta = 0.632$ ${\mathscr {T}}_E$ = 3.16 ${\mathscr {T}}_\Omega = 3333$ ${\mathscr {T}}_\Gamma = 250$
3	$\alpha = 2$ $\Omega = 0.01$ $\Gamma = 1$	$\eta _0 = 0.01$ $S_0 = 0.99$ $E_0 = 0$ $I_0 = 0.01$	$\alpha / \Gamma = 2$ $S_f = 0.193$ $S_\eta = 0.97$	${\mathscr {T}}_\eta = 0.99$ ${\mathscr {T}}_E$ = 6.34 ${\mathscr {T}}_\Omega = 100$ ${\mathscr {T}}_\Gamma = 1$

### Behaviour of the SEIR-C system

For the full SEIR-C system, in Case 1 (see the red solid line in Fig. 4a) initial exposures due to the initially present airborne infectious material occur rapidly, relative to the other processes; as expected from the relatively small value of ${\mathscr {T}}_\eta$ compared to the other timescales. However, these initial exposures will be relatively few in number since it follows that, from (30), the number of exposures ultimately arising due to the initially present infectious material is small, and hence $S_\eta \approx S_0$. After this short period of initial exposures, exposures will then be driven by infectious material emitted both by those initially infected, and those who have been exposed, incubated and become infectious during the outbreak, since the incubation timescale ${\mathscr {T}}_\Omega$ and the exposure timescale ${\mathscr {T}}_E$ are of the same order of magnitude. Finally, the removal timescale ${\mathscr {T}}_\Gamma$ is large relative to the exposure timescale and so it is expected that the whole population will be exposed before a significant amount of removal has taken place, i.e. the entire population will ultimately have become infected.

In Case 2 (see the red solid line in Fig. 4b), a significant number of exposures occur rapidly since the concentration of infectious material is large compared with the quasi-steady state, i.e. $\eta _0 \gg I_0$. This is to be expected based on (30), which indicates many exposures due to the initially present infectious material, combined with the fact that the timescale ${\mathscr {T}}_\eta$ is small relative to all other timescales. In this case, the incubation timescale ${\mathscr {T}}_\Omega$ and the removal timescale ${\mathscr {T}}_\Gamma$ are both large relative to the initial-exposure, ${\mathscr {T}}_\eta$, and production-exposure, ${\mathscr {T}}_E$, timescales, so that a large proportion of the population is expected to become exposed as a result of the initially present infectious material, or infectious material emitted by the initially present infectious population.

In Case 3, for the full SEIR-C system (see the red solid line in Fig. 4c), there appears a short period of initial exposures, before the initially infectious are rapidly removed, followed by a more prolonged period of outbreak as those exposed incubate slowly, are infectious for a limited amount of time, before then being rapidly removed. The result is a long-duration outbreak in which a significant fraction of the population remain ultimately uninfected.

### Comparison to existing models for outbreaks of airborne disease

In both Case 1 and Case 2, the SEIR-C system shows the number of susceptibles ultimately reaches zero, due to the large ratio of production-dilution parameter to the removal rate, as expected from the analysis in “The SEIR-C system at large time”)—this behaviour is accurately reflected in all three of the other airborne infection models. In Case 3, the ratio of production-dilution parameter to the removal rate is smaller, with $\alpha /\Gamma =2$, and within the SEIR-C system there remains a significant fraction of the population that are ultimately not infected; of the other three models, only the SEIR-QSS model correctly captures this important behaviour.

Both the Gammaitoni-Nucci and Wells-Riley models give a poor estimate of the expected outbreak behaviour in Case 1, as seen from the significant divergence between the full SEIR-C system and the blue lines in Fig. 4a. This is because the incubation timescale ${\mathscr {T}}_\Omega$ and the exposure timescale ${\mathscr {T}}_E$ are of a similar order of magnitude, so that some of the initially exposed population will incubate and become infectious while a significant number of susceptibles still remain; this drives a phase in which the outbreak spread is more rapid than either of the Gammaitoni-Nucci or Wells-Riley models can capture, leading to their significant underestimate exposure rate. Further comparison of the full system with the SEIR-QSS model also shows a difference in the predicted number of susceptibles, but the difference remains small (and is only visible during $6 \lesssim \tau \lesssim 20$); this difference never exceeds 0.04 consistent with the analysis in “The quasi-steady-state assumption” since $\max (\Gamma ,\Omega ) = 0.04$.

In Case 2, the SEIR-QSS model provides a poor prediction of the outbreak because the initial conditions are far from the quasi-steady state. Although it has been shown that the full SEIR-C system will approach a quasi-steady-state condition at an exponential rate, in this case to within a small margin ($0.004 = \max {(\Omega ,\Gamma )}$), the initially present infectious material results in a significant deviation in the number of susceptibles predicted in the SEIR-C system from that predicted in the SEIR-QSS model. A similar observation may be made between between the Gammaitoni-Nucci model and the Wells-Riley model in this case, with the Gammaitoni-Nucci model following SEIR-C and Wells-Riley following SEIR-QSS.

In Case 3, the Wells-Riley, Gammaitoni-Nucci and SEIR-QSS models all deviate significantly from the SEIR-C system. For the Wells-Riley and Gammaitoni-Nucci models, this is due to the relatively low value of the removal timescale, i.e. rapid removals influence the system significantly throughout the outbreak. The deviation between the SEIR-QSS model and SEIR-C system in this case is similarly due to large removal rate. Mathematically, *S* between the two systems may diverge within the margin $\max {(\Omega ,\Gamma )}=1$, which is significant.

### Considering the SEIR-C system using parameters from a real outbreak

We now apply our analysis of the SEIR-C system to a known outbreak, in order to predict the timescales over which the stages of such an outbreak occur, and to demonstrate how the selection of an appropriate outbreak model may be assisted by our understanding of the SEIR-C system. It has been noted (in the case of the COVID-19 pandemic) that a small number of ’superspreader’-type environments account for the majority of infections and so we apply our analysis to parameters associated with such a superspreader event^30,34^.

For this purpose, the Skagit Valley choir COVID-19 outbreak was chosen, as the parameters of the outbreak used in the SEIR-C system were well-documented^45^, with the exception of the incubation and removal rates as the outbreak occurred over only a 2.5 hour period. The typical incubation and recovery periods for COVID-19 have been extensively documented elsewhere, however^46,47^.

A best case for a similar outbreak is considered, in which the lower values of pulmonary breathing, incubation and quanta generation rates are used, along with the upper values of air change, decay, deposition and recovery rates, alongside the opposite as a worst case. The dimensional values of these are shown in Table 3, and the timescales and other properties that can be derived from theory in Table 4. The initial condition is based on a single infectious individual and 60 susceptibles, and an assumption of no infectious material initially present, as is believed to be the case in the Skagit choir outbreak.

In both the best case and worst case scenarios, $\alpha /\Gamma>> 1$, leading to a prediction that all susceptibles present will become infected over a sufficiently long time period. This may also be demonstrated by the solution of the implicit equation (26).

**Figure 5:**
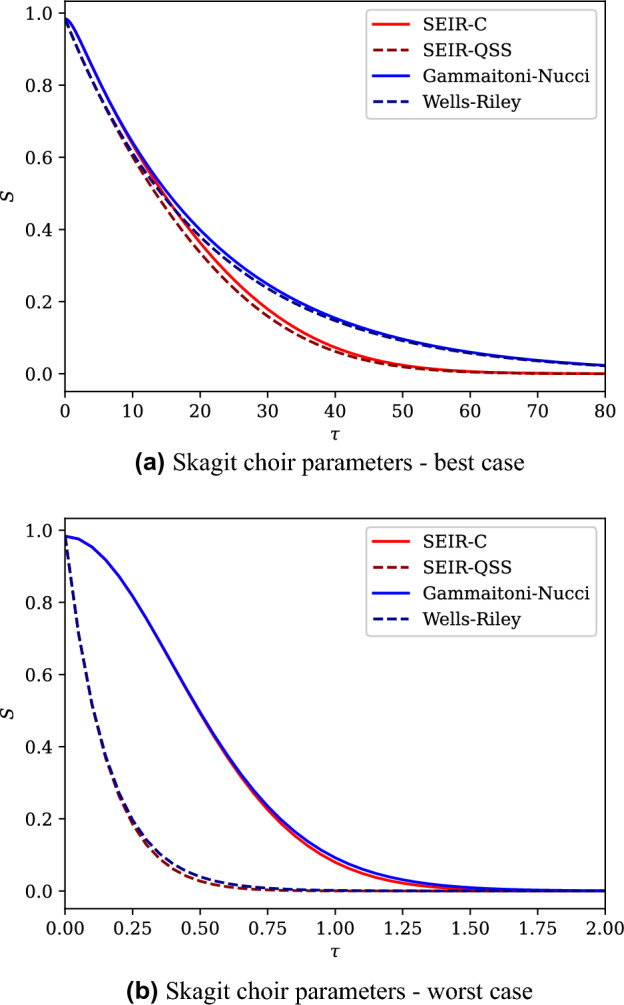
The value of *S* as a function of dimensionless time for the two example outbreak cases relating to the Skagit choir COVID-19 outbreak described in Table 3.

In both the best case and worst case scenarios for such an outbreak, the production-exposure timescale is far smaller than either the incubation or removal timescales. This, along with the large $\alpha /\Gamma$ ratio suggests that the entire susceptible population is likely to be infected by the initial infector before any of the susceptible population become infectious themselves. This can be used to justify neglecting incubation or removal when modelling such an outbreak, regardless of the duration of the outbreak investigated. This may be seen in Fig. 5, where in either case those models which incorporate incubation and removal do not significantly differ from those that do not.

In the best case scenario, although the initial condition does not meet the quasi-steady-state condition, the models under which the quasi-steady-state condition is maintained do not significantly deviate from those where it is not – recall that in the SEIR-C system, $\eta$ approaches the quasi steady state condition in exponential time to within an interval defined by the dimensionless incubation and removal rates.

In the worst case scenario, there is a significant difference between the results for the SEIR-C and SEIR-QSS models (and between the Gammaitoni-Nucci and Wells-Riley models). This may be understood by consideration of the production-exposure timescale ${\mathscr {T}}_E$, and recalling that within the dimensionless system the dilution timescale is defined as unity. In this case, the exposure timescale is short relative to the dilution timescale, so much of the exposure stage takes place before the quasi-steady-state condition is approached. A model which enforces the quasi-steady-state condition will therefore be inaccurate.

It has been previously noted that overdispersion is common in epidemiological modelling, due to the large variability in the infectiousness of individuals^30^. This may also be studied using the SEIR-C system by modelling the quanta emission rate according to a known distribution and performing Monte Carlo simulations and considering the ensemble result.

**Figure 6:**
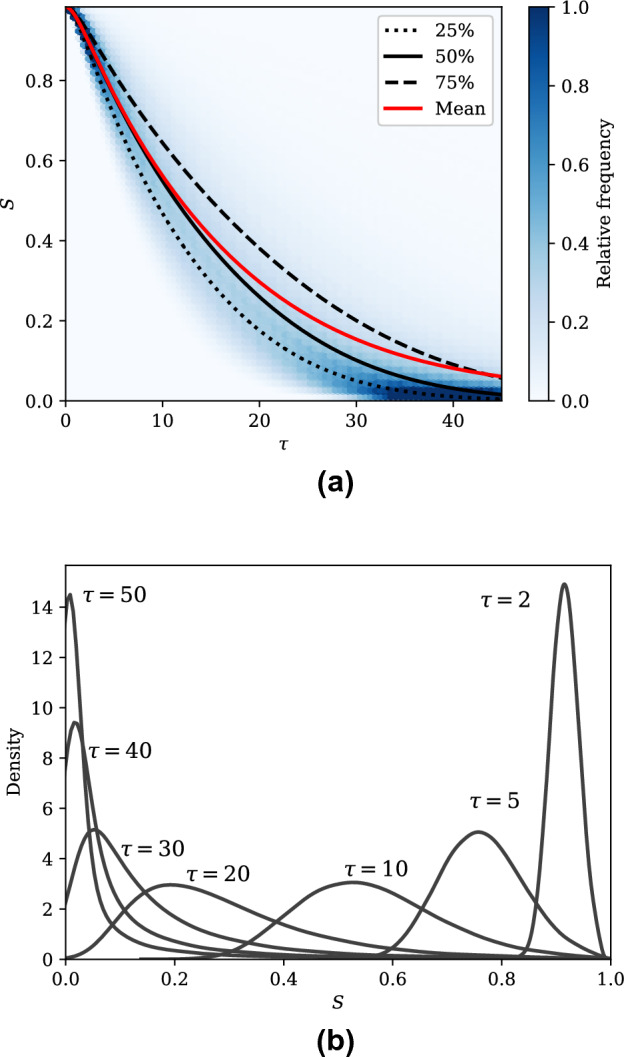
(**a**) The relative frequency distribution for *S* across 100,000 realisations of the SEIR-C system using the parameter set associated with the best case Skagit choir outbreak data seen in Table 3, with the quanta emission rate normally distributed about a mean of 970 quanta/h with a standard deviation of 390 quanta/h, and (**b**) the probability density functions of *S* at selected values of $\tau$ for the same case.

Figure 6 shows the evolution of *S* across 100,000 realisations for the quanta emission distribution associated with the Skagit choir outbreak, that is *q* normally distributed with a mean of 970 quanta/h and a standard deviation of 390 quanta/h. In this case, all other parameters correspond to the best base as seen in Table 3 so that the effect of variations in quanta emission rate may be seen in isolation, though they may of course similarly be varied. In Fig. 6a, the relative frequencies of *S* with respect to dimensionless time are shown, normalised against the total number of realisations. The 25th, 50th and 75th percentiles are also shown, showing that the majority of outbreaks inhabit a relatively narrow region about the mean result. Figure 6b shows the probability density function of *S* at different values of $\tau$ for the same case. It may be seen that the variance in distribution increases with respect to time before falling again at large time. Notably, for higher values of $\tau$ a significant positive skew emerges in the distribution, so that it may be seen that many outbreaks progress at a significantly slower rate than the mean, but few at a significantly faster rate. These slower outbreaks occur as a result of the inverse-square-root nature of the dominant production-exposure timescale with respect to the production-dilution parameter.

**Table 3:** Ranges of properties relevant to the SEIR-C model from the Skagit choir COVID-19 outbreak^45^.

Parameter	Symbol	Range	Units
Pulmonary breathing rate	*p*	0.65–1.38	m$^3$/h
Quanta generation rate	*q*	580–1360	h$^{-1}$
Deposition rate	$\lambda _k$	0.3–1.5	h$^{-1}$
Decay rate	$\lambda _d$	0–0.63	h$^{-1}$
Room volume	*V*	810	m$^{3}$
Ventilation rate	*Q*/*V*	243–810	m$^3$h$^{-1}$
Incubation rate	$\omega$	0.0028–0.042	h$^{-1}$
Removal rate	$\gamma$	0.0021–0.0083	h$^{-1}$

**Table 4:** The parameters and initial conditions defining two outbreak cases derived from data relating to the best- and worst-cases for the Skagit choir COVID-19 outbreak, along with key values that can be derived from these, and the timescales that result from these in the SEIR-C system.

Case	Parameters	Initial condition	Derived values	Timescales
Skagit choir equivalent – best case	$\alpha = 4.85$ $\Omega = 0.00067$ $\Gamma = 0.00265$	$\eta _0 = 0.0$ $S_0 = 0.984$ $E_0 = 0$ $I_0 = 0.016$	$\alpha /\Gamma = 1827$ $S_f = 0$	${\mathscr {T}}_E$ = 3.55 ${\mathscr {T}}_\Omega = 1490$ ${\mathscr {T}}_\Gamma = 377$
Skagit choir equivalent—worst case	$\alpha = 124$ $\Omega = 0.0134$ $\Gamma = 0.00067$	$\eta _0 = 0.0$ $S_0 = 0.984$ $E_0 = 0$ $I_0 = 0.016$	$\alpha / \Gamma = 185469$ $S_f = 0$	${\mathscr {T}}_E$ = 0.7 ${\mathscr {T}}_\Omega = 74.5$ ${\mathscr {T}}_\Gamma = 1490$

## Conclusions

Commonly used models for the spread of airborne infectious disease in (well-mixed) indoor spaces have been shown to be limiting cases of a combined quanta conservation and compartmental epidemiological system, herein described as the ‘SEIR-C system’. This system is analysed to provide insight into existing models and indoor airborne outbreaks more generally. By extending methods used previously for the SEIR model^8^, an implicit solution provides the final fraction of the population that will ultimately remain unaffected (susceptible) within the SEIR-C system. Four key timescales within the SEIR-C system were identified which combine to significantly influence the evolution of an outbreak, and their impact was discussed and illustrated.

The tendency for the quasi-steady-state assumption to give a good approximation to the full the SEIR-C system has been investigated. The quasi steady state is approached exponentially, to within an interval defined by the dimensionless removal rate and the dimensionless incubation rate. This highlights that if both of the dimensionless removal and incubation rates are small relative an operationally-defined tolerance, the quasi-steady-state approximation will be satisfied to within that tolerance in exponential time.

Comparison of the predicted outbreak behaviour under the SEIR-C system was compared to the widely used Wells-Riley, Gammaitoni-Nucci, and SEIR models for three distinct cases of parameter sets and initial conditions. It was shown that appreciation of analysis presented for the SEIR-C system, including the relevant timescales, enabled understanding and prediction of why, and when, each of these simpler outbreak models will provide a reasonable approximation to the full SEIR-C system, and when they will not. This approach was then applied to a parameter set associated with a well-documented COVID-19 airborne outbreak, and it was shown how variations in individual infectiousness may affect the progress of an outbreak and how this may be better understood via the SEIR-C system.

The selection of an appropriate outbreak model often requires a number of assumptions about the population and environment that may not be entirely realistic — for example, that the whole population remains present for the whole duration of an outbreak. By giving deeper insight into the stages of an airborne outbreak and the timescales associated with them, the methods presented here can be used as to when these assumptions will be reasonable. It is hoped that with further extension, the SEIR-C system may be used to develop models which encompass a wider range of indoor spaces in contexts which reflect their current usage. Obvious candidates include extensions to consider spaces such as open-plan offices and school classrooms which are typically regularly attended by the same population, who periodically leave and returns to the space, over long periods. It is also hoped that the methods presented here may be applied in more depth to stochastic models, for example to better account for the extreme variations in concentration of infectious material that can lead to overdispersion when modelling outbreaks.

## Data Availability

All data generated or analysed during this study are included in this published article.
